# Conservation Genetics of the Endangered Danube Clouded Yellow Butterfly *Colias myrmidone* (Esper, 1780) in the Last Central European Stronghold: Diversity, *Wolbachia* Infection and Balkan Connections

**DOI:** 10.3390/insects16020220

**Published:** 2025-02-17

**Authors:** Aleksandra Gwiazdowska, Robert Rutkowski, Marcin Sielezniew

**Affiliations:** 1 Museum and Institute of Zoology, Polish Academy of Sciences, Twarda 51/55, 00-818 Warsaw, Poland; agwiazdowska@miiz.waw.pl (A.G.); rrutkowski@miiz.waw.pl (R.R.); 2 Laboratory of Insect Evolutionary Biology and Ecology, Faculty of Biology, University of Bialystok, Ciołkowskiego 1J, 15-245 Białystok, Poland

**Keywords:** butterfly conservation, *COI*, *Colias myrmidone*, DNA barcode, *EF-1α*, genetic impoverishment, restricted gene flow, *Wolbachia*

## Abstract

The Danube Clouded Yellow (*Colias myrmidone*) has experienced one of the most dramatic declines among European butterflies. To estimate genetic diversity in the last population in Poland that has survived in the Knyszyn Forest (KF), we analyzed mitochondrial (*COI*) and nuclear (*EF-1α*) polymorphisms in individuals sampled in 2014 and 2022. The results were compared with genetic data obtained in 2014 from a recently extirpated nearby population (Czerwony Bór, CB). Because mtDNA polymorphisms in insects can be modulated by endosymbionts, the samples were screened for *Wolbachia*. The polymorphism of *EF-1α* indicated that diversity was gradually decreasing. The KF experienced rapid demographic processes, manifested by a significant change in allele frequency. The small differentiation in nuclear markers between the KF and CB in 2014 suggests that the regional population used to be genetically uniform. Four *COI* haplotypes that were identified in this study probably belong to two different haplogroups. *Wolbachia* was detected only in individuals with one specific haplotype, and the prevalence was female-biased, suggesting the induction of two reproductive manipulations. The most common *COI* haplotype found in Poland was the same as that reported from other parts of Europe, not only for *C. myrmidone* but also *C. caucasica.* These results allow us to question the distinctiveness of each taxa.

## Simple Summary

The Danube Clouded Yellow (*Colias myrmidone*) is a species that is under special conservation concern in Europe. The reasons for its quick and dramatic decline and local extirpations are not fully understood; genetic data are vital to understanding these reasons. We studied the last two neighboring populations from Central Europe—one that was recently extirpated and another that still exists—using a mitochondrial barcoding marker and a nuclear gene. We also tested samples for the endosymbiotic bacterium *Wolbachia,* which is known to induce reproductive manipulations in their hosts, affecting mtDNA diversity among populations. The results of our research suggest that a relatively high genetic polymorphism has decreased in recent years. Restricted gene flow that is related to habitat fragmentation and isolation has led to the quick differentiation of populations; however, these populations probably used to be genetically uniform, at least at the regional scale. DNA barcode-based analysis revealed the presence of two haplogroups, but, interestingly, only individuals from one of them were infected by *Wolbachia.* This phenomenon turned out to be female-biased, which has a complex impact on the host. Finally, a comparison of our data with other available data indicated significant barcode sharing between *C. myrmidone* and its sibling *C. caucasica,* which allows us to question the distinctiveness of both vulnerable taxa.

## 1. Introduction

The Danube Clouded Yellow *Colias myrmidone* is an example of one of the most dramatic declines in population among all European butterflies. The beginning of the century coincided with the process of the rapid extinction of the species. Van Swaay and Warren [1] evaluated its status as “Vulnerable” at the continental scale; however, only 20 years later, the weighted Red List value index in Europe put it among the top five most endangered species [2]. The butterfly is critically endangered in the European Union [3], being considered as a species of conservation concern due to its presence in annexes of the Habitats Directive. It was extirpated in several EU countries and has recently been confirmed to now only be found in Poland [4] and Romania [5].

The need to protect this species was first highlighted in 1990 in Bayern (Germany) [6]. The causes of the species decrease are complex and not fully understood. Some habitats deteriorated or ceased to exist as a consequence of agricultural intensification or due to the abandonment of grazing, as well as afforestation and changes in woodland management. Changes in water balance and the system of habitats have also been reported [4,7,8,9,10,11]. However, the butterfly has also disappeared from biotopes, i.e., with a large presence of larval food plants. The last populations have survived in fragmented landscapes, and recent studies have indicated that *C. myrmidone* requires a relatively large space and well-connected habitat patches [4]. Moreover, it is considered to be a high-risk species in the context of climate change [12], so some sudden local disappearances can be explained by unfavorable seasonal weather conditions [4].

Spatially isolated populations are particularly vulnerable to the effects of various factors that can cause extirpation [13]. Although the role of genetic factors in the extinction of populations and species is still a controversial issue and a hotly debated topic [14,15,16], there is no doubt that in isolated populations, genetic variability is reduced as a result of genetic drift and the increasing relatedness of individuals [17]. In turn, it is known that genetic variability is crucial for maintaining the adaptive potential of populations and species in the face of changing environmental conditions [18]. There are many examples of narrowed genetic diversity in butterfly populations threatened with extinction [19]. Low diversity and high differentiation are typical of sedentary specialists. On the other hand, it has been considered that intermediate species between generalists and specialists (like *C. myrmidone*) can suffer even more from genetic impoverishment when the gene pool cannot be refreshed due to a lack of connectivity [20]. It is also known that changes in the level of genetic diversity can be a valuable clue when identifying populations that should be classified as endangered [21]; this approach is applicable in the case of butterflies [22]. Therefore, estimating the level of genetic variability and tracking its changes may prove crucial in planning conservation strategies for endangered species [23,24].

Being a large and spectacular butterfly, *C. myrmidone* remains, in some respects, an insufficiently studied species. Taking into account the genetic aspect, there exist taxonomic studies of the genus *Colias* or higher taxonomic units based on analyses of single individuals from some species within the genus. In particular, genomic studies have provided knowledge on the evolution of *Colias* [25]. However, population studies of *C. myrmidone* have not been conducted to date. Therefore, there are significant gaps in the knowledge regarding, for example, genetic structure. Understanding genetic differentiation, diversity, and other ongoing processes is vital from a conservation point of view; without such information, it is hard to imagine, for example, possible reintroduction efforts [26]. The majority of available data on *C. myrmidone* are in relation to mtDNA, namely the cytochrome c oxidase subunit I (*COI*), a section of which has become the standard DNA barcode for animals [27] and has been added to barcoding libraries [28,29].

The aim of the present study was to obtain insights into the last regional population inhabiting the Knyszyn Forest in Northeastern Poland. Over the last thirty years, the species has disappeared from the entire country, except for in this isolated area [4]; the next nearest known localities are in Southern Belarus [30]. Hence, we carried out extensive sampling in the Knyszyn Forest and obtained some material from the recently extirpated penultimate population in the same region. We analyzed *COI* and the nuclear gene elongation factor 1α (*EF-1α*), which is commonly used in phylogenetically oriented butterfly surveys to complement mtDNA markers [31,32].

Analyses of *COI* sequences belonging to over 300 butterfly species in Western Europe indicate that the spatial differentiation in mtDNA is negatively correlated with species traits determining dispersal capability and colonization ability [33]. However, variation in mtDNA may not be correlated with genomic variation due to inheritance mechanisms and other factors, e.g., microbiological infections. Hence, we tested samples for the presence of α-proteobacteria *Wolbachia* (Rickettsiaceae), i.e., maternally inherited symbiotic bacterium of ca. two-thirds of all arthropod species [34], which can affect the reproduction of its host [35].

The consequence of segregation distortion may be the origin of different mtDNA lineages or the reduction in genetic diversity when a single haplotype (or haplogroup) dominates a population/region/range [26,36,37]. *Wolbachia* infections may affect the mitochondrial structure of hosts [38] and even mimic cryptic speciation [39]. In the case of species of conservation concern, this bacterium is a possible serious threat since the introduction of *Wolbachia* into uninfected populations or the introduction of a new strain may increase the risk of extinction. Therefore, knowledge about the pattern of infection is vital in the context of actions that incorporate captive breeding or translocation [40,41].

## 2. Materials and Methods

### 2.1. Study Species

**Figure 1:**
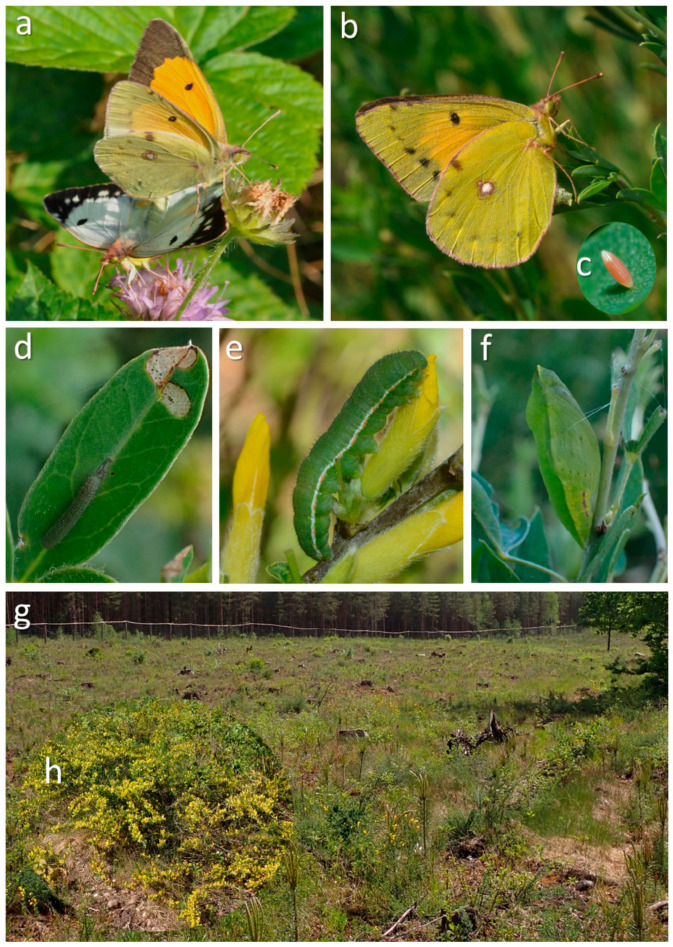
*Colias myrmidone* and its habitat in the Knyszyn Forest: (**a**) a mating pair with a gynomorphic (alba) female (below), (**b**) an ovipositing andromorphic (orange) female on the only local larval food plant, i.e., *Chamaecytisus ruthenicus*, (**c**) an egg, (**d**) a young caterpillar, (**e**) a full-grown caterpillar, (**f**) a chrysalis, (**g**) a typical patch of habitat, i.e., recently replanted clear-cut, (**h**) a flowering *Chamaecytisus ruthenicus* plant in the spring; © Marcin Sielezniew.

The Danube Clouded Yellow *Colias myrmidone* (Esper, 1781) is a relatively large pierid butterfly (Lepidoptera, Pieridae), with a wingspan of about 40–46 mm. Sexual dimorphism is distinct, with two main female forms, i.e., gynomorphic orange and andromorphic pale yellow. Two or three generations are on the wing every year, depending on season and locality. A variety of flowers are used as nectar resources. Males are patrollers; they move in fast flight looking for mates. Females lay eggs singly on the upper side of the leaves of several *Chamaecytisus* species. Caterpillars initially leave window-like traces of feeding; later, they eat whole leaves. Pupation takes place on food plants or vegetation close to the ground (Figure 1). The medium-sized larvae of the first generation overwinter among fallen leaves of the food plant [4].

The distribution range of *C. myrmidone* is almost entirely limited to Europe. A dramatic decline has been observed across the continent with extinctions in Germany, Czech Republic, Hungary, Austria, Slovenia, and Lithuania, and the butterfly has been reported recently only from Poland, Romania, Belarus, Ukraine, and Russia [9]. In Balkans, *C. myrmidone* is replaced by a sibling taxon *Colias caucasica balcanica* Rebel, 1901, whose status is, however, still disputable [29].

**Figure 2:**
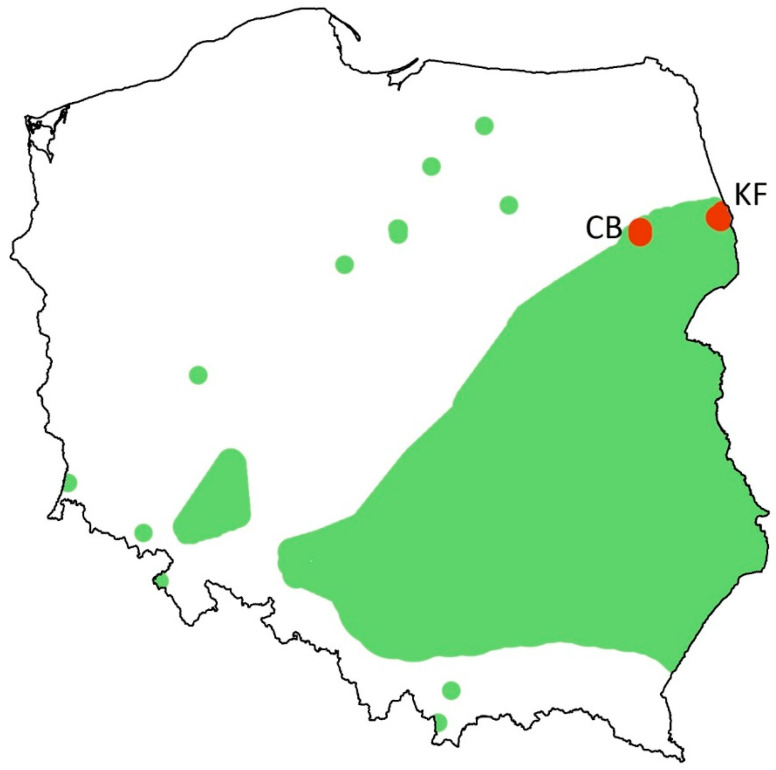
Map of Poland, showing sampling sites (red dots) at the background of the former distribution range (green patches) and isolated sightings (green dots) of *Colias myrmidone*. KF—the last population in the Knyszyn Forest, CB—Czerwony Bór, where the butterfly was extirpated around 2019 (based on [42], modified).

A severe contraction of its range has been observed in Poland, where the species at present occurs only in the Knyszyn Forest in the northeastern part of the country (Figure 2) [4,42]. The last metapopulation depends on forestry, and almost all its habitats are recent clear-cuts. The only larval food plant available, i.e., *Chaemocytisus ruthenicus*, is present in some tree stands, and after logging, it typically grows lush and covers the ground extensively in some patches due to increased sun exposure. Therefore, clear-cuts may be colonized by *C. myrmidone* and support local populations for several years until an increase in shading is caused by growth of replanted trees or natural succession [4]. The penultimate locality was the former military area (Czerwony Bór) situated ca. 100 km away (Figure 2), where *C. myrmidone* was extirpated around 2019, although the habitat looked still suitable [4].

### 2.2. Sampling for Genetic Studies

The main sampling was performed in 2022 in the Knyszyn Forest along intensive mark–release–recapture studies (see [4]). A total number of 125 individuals was sampled, including 53 females (28 andromorphic and 25 gynomorphic) and 73 males. Sex was recorded for all individuals, and for females, color forms were also recorded (Table S1). Additionally, we used samples collected in the Knyszyn Forest (12) and in Czerwony Bór (21) in 2014, but details concerning sex were not recorded at that time. All tissue samples were taken using a nonlethal method. Small fragments of hind wings (~2 mm^2^) or middle legs were torn off using tweezers and then individually stored in 95% ethanol. Pierid butterflies (contrasting to nymphalids) use all six legs for walking, so such damage is insignificant. No approval of research ethics committees was required for such procedure in Poland since sampling was conducted with unregulated invertebrate species. Here, it is worth mentioning that sampled individuals were often recorded again during mark–release–recapture studies [4]. The lack of negative effects of single-leg removal on the survival, behavior, and mobility of individuals has also been confirmed for other butterfly species [43].

### 2.3. Laboratory Procedures

DNA was extracted from a single leg or a single piece of wing. In the case of fresh samples (collection 2022), the GeneMATRIX Bio-Trace DNA Purification Kit was used, whereas for older sample specimens (2014), we used the GeneMAGNET Human and Animal Tissue DNA Purification Kit (EURx, Gdańsk, Poland). The fragment of wing or leg was cut into pieces in a sterile Petri dish using a sterile scalpel. The tissue fragments were placed in tubes with lysing buffer with Proteinase K and incubated overnight at 56 °C. Then, extraction of DNA was performed according to a standard protocol. DNA extracts were stored at −20 °C for further procedures.

To estimate genetic diversity, we analyze fragments of mitochondrial *COI* (*Cytochrome Oxidase Subunit 1*) and nuclear *EF-1α* (*Elongation Factor-1α*) genes. We aimed to amplify and sequence ~658-base pair (bp) fragment of *COI* and ~650 bp of *EF-1α*. Additionally, we amplified fragments of Wsp (*Wolbachia surface protein*) gene (~540 bp) to detect presence of endosymbiotic *Wolbachia*. In the case of *COI*, PCR amplification was carried out using primers LCO1490 (forward) and HCO2198 (reverse) [44]. Fragments of *EF-1α* were amplified using primers ELF2 (forward) [45] and efrcM4 (reverse) [46]. To detect *Wolbachia*, we used primers wsp81F (forward) and 691R (reverse) [47], amplifying the fragment of Wsp gene.

In the case of *COI* and *EF-1α* the reaction mixture contained 2 or 3 μL of DNA extract depending on the initial concentration (ranging from 10 to 100 ng/μL), 7.5 μL or 8.5 μL of water (depending on the amount of DNA), 12.5 μL of PCR Master Mix (Promega GoTaq G2 Hot Start Green Master Mix) and 1 μL of forward and reverse primer. The amplification conditions for *COI* were as follows: initial denaturation for 7 min at 94 °C, 35 cycles of 1 min at 94 °C, 1 min at 54 °C, 2 min at 72 °C and final elongation for 7 min at 72 °C. For EF1α, we used the following PCR conditions: initial denaturation for 5 min at 94 °C, 40 cycles of 1 min at 94 °C, 1 min at 62 °C, 1 min 30 s at 72 °C and final elongation for 10 min. at 72 °C. Testing presence of *Wolbachia*, the following PCR conditions were applied: initial denaturation for 5 min at 94 °C, 40 cycles of 1 min at 94 °C, 1 min at 53 °C, 1 min at 72 °C and final elongation for 10 min at 72 °C.

Agarose gel electrophoresis was performed to visualize the PCR products. In the case of no visible bands, re-amplification was performed. Namely, as a template for PCR reaction, we used 2 μL of previous PCR final mixture. In addition, the reaction mixture contained 8.5 μL of water, 12.5 μL of PCR Master Mix (Promega GoTaq G2 Hot Start Green Master Mix), and 1 μL of forward and reverse primer. The amplified fragments were sequenced using the ABI 3500XL Genetic Analyzer. The PCR products were purified using ExoSAP-IT (Applied Biosystems, Waltham, MA, USA), following the standard protocol. Next, we performed sequencing PCR using the BigDye Terminator v3.1 cycle sequencing kit (Applied Biosystems). The reaction mixture contained 2 μL of template, 4 μL of BigDye Terminator, 2 μL of BigDye terminator sequencing buffer, 2 μL of forward or reverse primer (3.2 μM), and 10 μL of water. The last stage before sequencing was purifying PCR products using HighPrep DTR (MAGBIO) following standard protocol. The results were sequenced using the ABI 3500XL Genetic Analyzer.

### 2.4. Statistical Analysis

DNA sequences were aligned in BioEdit v 7.0.4 [48] and revised manually. The haplotype reconstruction of the nuclear *EF*-1 α gene from unphased/genotype data was conducted using the algorithms provided in PHASE as implemented in DNASP 5.0 [49].

To characterize the diversity of amplified genes, the number of haplotypes (*H*), haplotype diversity (*h*), nucleotide diversity (π), and mean number of nucleotide differences among the haplotypes (*k*) in the overall sample and both populations were calculated using DNAsp 5.10 [49]. ARLEQUIN v3.5.2.2 [50] was used to estimate genetic differentiation among the groups of samples (*Θ*_ST_), dividing by localization (Knyszyn Forest vs. Czerwony Bór) and year of sampling (2014 vs. 2022). Permutation test (1000 repetition) was applied to estimate significance of the genetic differentiation. 

To analyze whether previous fluctuations in population size had affected current genetic diversity, we used DNAsp and ARLEQUIN to perform basic neutrality tests, detecting past population expansion, Fu’s *F_S_* [51], and Tajima’s *D* [52]. Both tests use the infinite site model without recombination to test for departures from selective neutrality and population equilibrium for intraspecific data. Fu’s *F_S_*, Tajima’s *D,* and related statistics signal an excess of rare mutations when the values are negative and differ significantly from zero. Fu’s *F_S_* uses information from the haplotype distribution and has greater statistical power to detect population expansion than other available tests [51]. Low *F_S_* values indicate an excess of single substitutions, usually due to expansion.

Tajima’s *D*-test, in turn, compares the number of nucleotide differences between sequences and the number of differences between segregating sites. In this case, population expansion will result in a significant negative departure from zero [52].

The significance of both values was determined by way of a coalescent simulation, using 1000 replicates in DNAsp. Because Fu [51] showed that a significance of 0.02 was equivalent to a 0.05 level where *Fs* was concerned, the test was considered significant when *p* ≤ 0.02.

To identify past demographic processes that could have influenced the observed genetic diversity in *C. myrmidone* in Poland, a mismatch distribution analysis [53] was performed in ARLEQUIN. We compared distributions for numbers of nucleotide differences among haplotypes in sampling locations and in the whole Polish population of the species to the distribution of differences in a model population that had undergone recent expansion. In a stable population, the distribution is usually multimodal, whereas in a population following recent expansion, a unimodal distribution of differences may be anticipated. A thousand bootstrap replicates were used to generate an expected distribution using a model of sudden demographic expansion. The sum of squared deviations (*SSD*) between the observed and model distributions was used as a test statistic. Significant values for *SSD* indicate deviation from sudden expansion. Additionally, we estimated the raggedness index and its significance. This measure quantifies “smoothness” of the observed mismatch distribution. Low values for raggedness are typical for an expanding population, whereas higher values are expected in the case of stationary or bottlenecked populations [54].

For both genes, a median-joining haplotype network [55] was constructed in NETWORK v4.6.1.1. (Fluxus Technology Ltd., Suffolk, UK) and PopArt v4.8.4. (http://popart.otago.ac.nz, accessed on 12 December 2024). In the case of *COI*, haplotypes identified in *Colias caucasica* were also included (Supplementary Table S2), as the comparison of the obtained haplotypes with the sequences from GenBank showed that one of the *C. myrmidone* haplotypes (Cm*COI*-2) was identical to the haplotype found in another species―*C. caucasica*.

Finally, the *COI* haplotypes identified in this study were compared with homologous sequences for the *C. myrmidone* obtained from GenBank (https://www.ncbi.nlm.nih.gov/genbank, accessed on 12 December 2024) (Supplementary Table S2). To resolve the evolutionary relationship between haplotypes identified in this study and those found in other European *Colias* species, we constructed a phylogenetic tree using the neighbour-joining method, as based on Tamura-3 model with gamma distribution (T92+G). The Tamura model was selected in jMODELTEST [56], in line with the corrected Akaike information criterion (AICc); and the tree was inferred using MEGA 11.0 [57]. 

## 3. Results

### 3.1. Cytochrome Oxidase Subunit 1

We obtained a 587 bp fragment of the *COI* from a total of 159 *C. myrmidone* from Poland, including 137 (125 from 2022 and 12 from 2014) specimens from the Knyszyn Forest and 22 from Czerwony Bór (2014). This fragment was found to include 15 polymorphic sites (13 parsimony-informative sites). The variable sites represented 10 transitions and 5 transversions. The base composition of the *C. myrmidone COI* fragment was as follows: A—31.03%; C—37.06%; T—22.98%; G—8.93%. Among the 159 sequences obtained, we identified four haplotypes (Table 1, GenBank Acc. no: PV031351–54). However, just one of these proved clearly dominant in terms of the frequency of occurrence in Poland. This was Cm*COI*-1, which was found in 87% of the samples. This haplotype further corresponded to the haplotypes from GenBank that were previously identified in *C. myrmidone* (Table S2). Cm*COI*-1 was present in both investigated populations from Poland and was the only haplotype found in Czerwony Bór. Cm*COI*-2 was present in ≈12% of samples, and the remaining haplotypes (Cm*COI*-3 and Cm*COI*-4) were identified in single individuals from the Knyszyn Forest (Table 1).

The overall haplotype diversity was low, at 0.224 ± 0.041, with an associated nucleotide diversity of 0.00452 ± 0.00086. The average number of nucleotide differences among haplotypes (*k*) was 2.652 ± 0.023 (Table 1). We did not find any evidence for the recent expansion in the populations from Poland, as indicated by non-significant values of Fu’s *F_S_* and Tajima’s *D*. Similarly, significant *SSD* suggested a deviation from the sudden expansion model. The relatively high value of the raggedness index (*r*) may indicate that the studied population has passed the demographic bottleneck.

**Figure 3:**
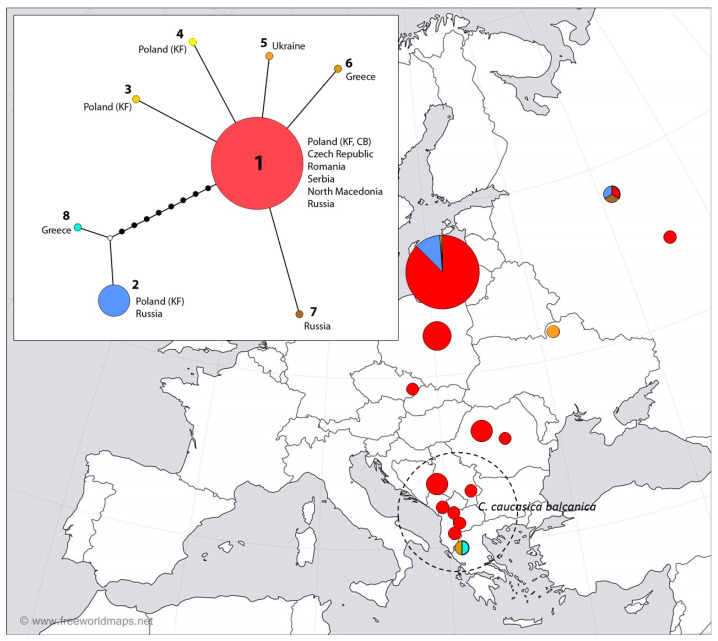
Distribution of *COI* haplotypes among populations of *C. myrmidone* in Poland (KF—the Knyszyn Forest, CB—Czerwony Bór) and different localities of *C. myrmidone* and *C. caucasica* within its range (sequences available in the GenBank database). Haplotype network reconstruction was drawn using the median-joining method. The sizes of the circles on the haplotype network are directly proportional to the number of individuals analyzed.

The median-joining network for haplotypes identified in this study (Figure 3) revealed one “core haplotype”, which referred to 163 individuals, including 139 individuals from the Knyszyn Forest and Czerwony Bór (Table S1) and 24 individuals from GenBank (Table S2), as well as several satellite, low-frequency haplotypes found in the Knyszyn Forest (Cm*COI*-3, Cm*COI*-4) and GenBank, i.e., single individuals of *C. caucasica* from Greece (Cm*COI*-6, MW501563.1) and *C. myrmidone* from Russia (Cm*COI*-7, MW501352.1), differing by single substitution. However, we also identified a second haplogroup consisting of Cm*COI*-2, which was found in the Knyszyn Forest, and the case of a single individual from Russia (MW499355.1), as well as a closely related haplotype, identified in *C. caucasica* from Greece (Cm*COI*-8, MW499440.1) (Table S2). Both haplogroups differed by nine substitutions.

**Figure 4:**
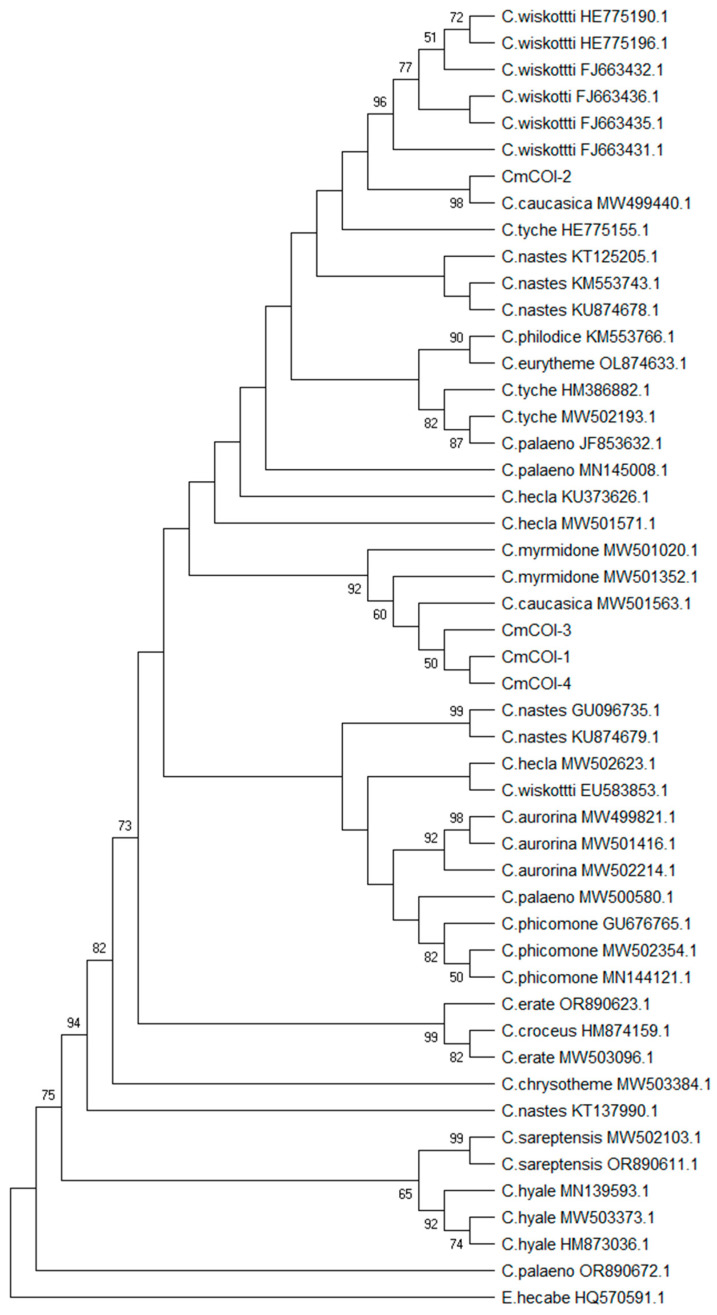
The phylogenetic tree of *COI* haplotypes, inferred using the neighbour-joining method. All haplotypes identified in this study (Cm*COI*-1 to Cm*COI*-4) were included together with homologous sequences of European *Colias* sp. from GenBank. The optimal tree is shown. The percentage of replicate trees in which the associated taxa clustered together in the bootstrap test (500 replicates) are shown next to the branches, but only values over 50% were displayed. The tree is drawn to scale, with branch lengths in the same units as those of the evolutionary distances used to infer the phylogenetic tree. The evolutionary distances were computed using the Tamura 3-parameter method, as described in Material and Methods*. COI* sequence of *Eurema hecabe* was used as an “out-group”.

The neighbour-joining phylogenetic tree (Figure 4) for the combined data set (novel haplotypes identified in this study and homologous sequences deposited in GenBank—49 haplotypes in total) revealed a surprising distribution of *C. myrmidone COI* haplotypes. Firstly, all sequences were grouped in a common clade with haplotypes of *C. caucasica*. Secondly, Cm*COI*-2, grouped with a *C. caucasica* haplotype from Greece, was located in a separated part of the phylogenetic tree, within a common clade with *C. wiskotti*. It should be noted that most nodes on the phylogenetic tree had low statistical support. However, the analysis highlighted the distinctiveness of the Cm*COI*-2 haplotype.

### 3.2. Elongation Factor EF-1α

The successful amplification of a nuclear gene fragment occurred in 151 of the 159 individuals analyzed. In the 581 bp fragment, 18 polymorphic sites were found, which resulted in the identification of 18 haplotypes (GenBank Acc. no: PV033841–PV033858). In the Knyszyn Forest population, we found 14 haplotypes, and *h* was 0.600 (Table 2). We found no differences in haplotype frequencies in the two largest *COI* haplogroups in the Knyszyn Forest (Cm*COI*-1 vs. Cm*COI*-2; *Θ*_ST_ = 0.003; *p* > 0.05), while the genetic differentiation between samples collected in 2014 and 2022 was clearly higher and statistically significant (*Θ*_ST_ = 0.08; *p* < 0.05). In 2022, there were more haplotypes, but the haplotype diversity was higher in samples collected in 2014 (Table 2). This suggests that a higher number of haplotypes was strictly interlinked with a higher number of samples. Similarly, we found a higher nucleotide diversity (π) and *k* in the 2014 samples than in the 2022 samples. This suggests that genetic diversity has been gradually decreasing in the Knyszyn Forest over recent years.

In the case of Czerwony Bór, among 16 analyzed samples, we found as many as 9 haplotypes and a very high corresponding allelic diversity (*h* = 0.934). Interestingly, there was no significant genetic differentiation between samples from the Knyszyn Forest, collected in 2014, and Czerwony Bór (*Θ*_ST_ = 0.019; *p* > 0.05), but we found a much higher and significant differentiation for comparison between Czerwony Bór and the Knyszyn Forest in 2022 (*Θ*_ST_ = 0.16; *p* < 0.01). This suggests that, over the last decade, there has been a significant change in the frequency of nuclear alleles in the Knyszyn Forest as a result of, for example, increased genetic drift in an isolated population.

Neutrality tests were non-significant in the majority of analyzed groups of sequences, although negative values suggested some signs of the historical expansion of the species in central Europe. Moreover, we found a negative and significant value of the Fu’s test for overall analysis (Table 2), indicating that the excess of allele numbers in the population of *C. myrmidone* from Poland was possibly due to their recent expansion. Interestingly, estimating neutrality tests in *COI* haplogroups (Cm*COI*-1 and Cm*COI*-2), we found a negative and significant value of Fu’s test (*F_S_* = −5.781; *p* < 0.01) for haplogroup Cm*COI*-1 but not for haplogroup Cm*COI*-2 (*Fs* = −2.308; *p* > 0.05). It should be noted, however, that a non-significant value of Fu’s *F_S_* could be interlinked with a low sample size (*N* = 17 for haplogroup Cm*COI*-2 vs. *N* = 105 for haplogroup Cm*COI*-1). We found low values of raggedness index *r* and non-significant *SSD*, suggesting that the studied nuclear marker preserves traces of demographic expansion.

**Figure 5:**
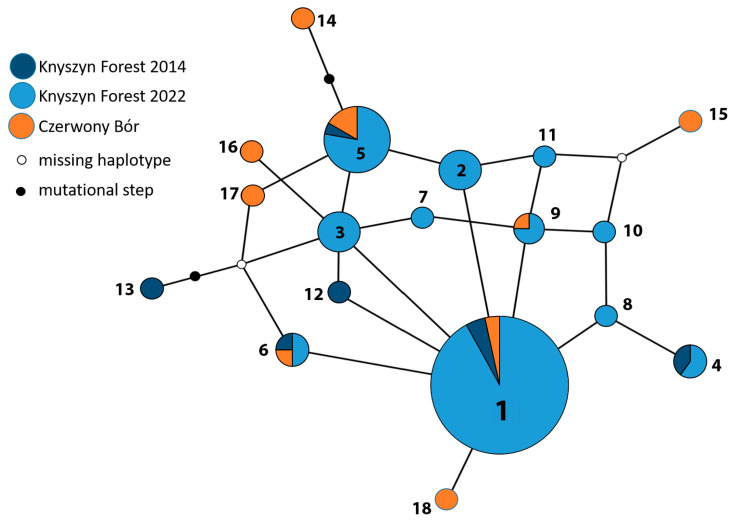
The median-joining network of 18 haplotypes was identified in this study. To simplify the picture, in the case of low-frequency haplotypes, the prefix “Cm*EF*” was omitted from the haplotype names. The size of a circle is approximately proportional to the frequency of occurrence of the given haplotype in the sample of 151 individuals. Each line connecting two circles represents a single substitution differentiating the haplotypes.

A comparison of the frequency of haplotype occurrence in both populations showed the presence of one dominant haplotype in terms of frequency haplotype—Cm*EF*-1—occurring in almost 60% of individuals. The second most frequent haplotype—Cm*EF*-5—was found in slightly over 12% of individuals. Both haplotypes differ by only two substitutions and occurred in both studied populations. In the Knyszyn Forest, Cm*EF*-1 was definitely dominant in terms of frequency, while in Czerwony Bór, it was one of the two most frequent haplotypes (Table 2). In general, the studied populations clearly differed in terms of the occurrence of the *EF*-*1*α haplotypes. Only four haplotypes were shared between both populations, while as many as fourteen haplotypes were unique to one or the other (Figure 5).

The haplotype network (Figure 5) was typical for *EF-1α*, indicating that the dominant haplotype—Cm*EF*-1—occupied the core position, while most of its satellite haplotypes differed by single substitutions and showed a low frequency. Additionally, many alternative linkages were found between the satellite haplotypes. A characteristic feature of this network is the presence of a second, relatively frequent haplotype elsewhere in the network. Cm*EF*-5 was also found in both populations and seems to be quite frequent in the Knyszyn Forest, although it occupies a distal position in the network.

### 3.3. Wolbachia Infection

To explore the prevalence of *Wolbachia* infection, *Wolbachia* DNA was detected in *C. myrmidone* samples via PCR amplification of the surface protein gene *wsp*. We tested 53 samples from the Knyszyn Forest, collected in 2022, including 26 males and 27 females. Only the two most frequent haplotypes were screened. We found that 30% of *C. myrmidone* in the Knyszyn Forest were positive for *Wolbachia*. Considering the sex of the infected individuals, we found 11 infected females and only 4 males. Considering the *COI* haplogroups, we found infected individuals only within the haplogroup Cm*COI*-2, and no infections were observed in the haplogroup Cm*COI*-1. The analysis of the sequence of the amplified fragment of the *wsp* gene indicated the presence of three haplotypes, differing by a single substitution. A comparison of the obtained sequences from the *Wolbachia* sequences deposited in GenBank indicated that the identified strain was not previously identified in the *Colias* species. Instead, two *Wolbachia* haplotypes identified in this study (Cm*Wsp*-1, Cm*Wsp*-2; GenBank Acc. no: PV085849–PV085850) exhibit a very high sequence similarity (99.82 and 99.86, respectively) with one of the *Wolbachia* strains identified previously in *Pseudophilotes bavius* (GenBank Acc. no: MK784199.1) [58].

## 4. Discussion

### 4.1. Genetic Diversity of the Last Remaining Polish Population

While analyzing the level of genetic variability in the mitochondrial *COI* gene in the Knyszyn Forest, we found the occurrence of four haplotypes (Cm*COI*-1–4), which is the same number as those found so far within the species [29]. Nonetheless, we should assume that haplotype and nucleotide diversity were rather low. Here, it should be noted that previous studies indicate that the diversity in *COI* in terms of the representatives of *Colias* Fabricius, 1807 across Europe, is relatively low compared to other butterfly genera [28,29,59]. This pattern can be explained to some extent by the short evolutionary history of the *Colias* genus, which evolved only ~3.5 Ma [25]. Nonetheless, the presence of four *COI* haplotypes in spatially and temporally (for at least 20 years) isolated populations seems surprising, especially considering that we found only one haplotype in the recently extirpated Czerwony Bór population. However, the identification of only one haplotype in Czerwony Bór, as well as in the Knyszyn Forest in 2014, may be related to the small sample from the population. It is unlikely that additional haplotypes appeared in the isolated Knyszyn Forest after 2014. It should be assumed that when analyzing only a few individuals, only the haplotype would be detected, which would definitely be dominant in terms of frequency.

A comprehensive data set of *C. myrmidone COI* sequences indicated the presence of two haplogroups, but the one with Cm*COI*-1 as the central haplotype is definitely more widespread. Among European butterflies, the star-shaped topology of the *COI* network (a low number of prevalent haplotypes and numerous, but of low frequency variants resulting from subsequent mutations) is the most typical [29], which can be seen in *Phengaris arion* [60]. In the case of clearly distinct lineages, the pattern can be different with several relatively equal clades, e.g., for *Parnassius mnemosyne* [61]. Cm*COI*-2 belonging to the separate haplogroup differed from Cm*COI*-1 by nine substitutions. Even higher intraspecific divergences have been reported for *Colias palaeno*, where most individuals were placed in the typical star-like network, but three haplotypes were clearly distinct (differing by up to as many as 50 mutations) and were likely the result of introgression from other *Colias* species [62]. For *C. myrmidone*, there is no indication of such a scenario, but the possibility of ancient hybridization cannot be completely ruled out.

The number of *EF-1α* alleles in the present study was clearly higher in both localities compared to the number of *COI* haplotypes. Divergent patterns of mtDNA and nDNA diversity are not uncommon among butterflies, and a higher sequence polymorphism in *EF-1α* than *COI* seems to be common [60,63,64,65,66]. Mitochondrial lineages exhibit a much faster lineage sorting rate, and the random elimination of haplotypes is much faster than the nuclear allele extinction rate; therefore, evolutionary relationships could be oversimplified [67].

Accordingly, we found a moderate genetic diversity in *EF-1α* (*h =* 0.60); thus, we found no evidence that isolation reduced genetic diversity, which may be surprising for such a highly endangered species. However, in butterflies, the level of genetic diversity has been shown to be unrelated to their abundance or natural history traits [68]. The relationship between population size and genetic variability is not straightforward. In general, there are examples of populations that exist despite low numbers and reduced genetic variability [69]. Conversely, there are known populations that have experienced significant declines despite having a rich gene pool [70]. Also, some populations maintain a higher-than-predicted heterozygosity and allelic diversity, although they are small and long-isolated [71]. In the case of the *Melitaea cinxia* metapopulation, the relationship between heterozygosity and extinction disappeared when demographic and environmental variables were introduced into the model analyses, indicating that the association between genetic diversity and extinction could be detected only under some conditions. For example, the rate of extinction does not decline with increasing heterozygosity in small populations, whereas such a pattern is observed in the case of large populations [22]. Hence, we can assume that our study population from the Knyszyn Forest could be another example of a small, isolated population, maintaining quite a high genetic diversity.

In the face of the complete lack of gene flow in recent years resulting from isolation [4], one possible explanation is that the current levels of genetic diversity reflect pre-disturbance values, and insufficient time has passed for a detectable reduction in genetic diversity. The population of *C. myrmidone* in Poland collapsed at the beginning of the 21st century, and the butterfly survived only in several isolated regions. In 2022, i.e., the year of intensive sampling, the metapopulation in the Knyszyn Forest was relatively large due to the previous enlargement of the habitat network being a consequence of intensive forestry, creating ephemeral patches [4].

The Knyszyn Forest is located in Northeastern Poland. For many animal species, including butterflies, a high genetic diversity on mtDNA was found here, which can be attributed to the secondary contact zone of lineages from different glacial refuges [72,73,74,75]; this is an example that contradicts the “southern richness, northern purity” paradigm. Secondary contact following postglacial recolonization and interspecific hybridization may be crucial in shaping the pattern of genetic diversity of populations in western Eurasia, especially in insects [76].

Nonetheless, the genetic diversity of the Knyszyn Forest population seems to have gradually decreased. Despite the lower sample size, we found clearly higher indices of genetic diversity in the group of specimens from 2014 than from the 2022 group. Moreover, genetic differentiation in *EF-1α* allele frequency was found between both periods. Similarly, mean values of genetic diversity indices for *EF-1α* were higher in Czerwony Bór, where samples were also collected in 2014. Nuclear data also suggest that the Polish population used to be genetically uniform, as we did not confirm significant genetic differentiation between the Knyszyn Forest and Czerwony Bór for samples from 2014.

According to the classification of Habel and Schmitt [20], *C. myrmidone* can be considered as an intermediate species between specialists and generalists. Such species often occur in large population networks and are characterized by a relatively high genetic diversity, which is sustained by gene flow. Population connectivity rescues genetic diversity after a demographic bottleneck [77] and a lack of interconnectivity due to the fragmentation, degradation, and isolation of habitats, which may lead quickly to inbreeding depressions contrasting to specialists. As a consequence, populations suffer from sudden genetic impoverishment and possibly reduced adaptability (see, e.g., [78]) and, therefore, may be at the highest risk of extinction even compared to specialists, for which a low genetic diversity is often typical. The fragmentation and isolation of breeding areas are considered as one of the main causes of the collapse of the Hungarian population [11]. Although our study suggests a depletion of the gene pool in the Knyszyn Forest, an analysis of genetic material from more distant periods is needed to confirm that *C. myrmidone* fits in with the predictions for intermediate species in terms of genetic diversity. The source of such specimens may be museums and private collections. Additional molecular markers, relying on short DNA fragments (e.g., microsatellites or single-nucleotide polymorphisms), would be more appropriate for often fragmented genetic material [79].

Indeed, in the past, *C. myrmidone* used to be considered a mobile species, but studies of the metapopulation in the Knyszyn Forest show that when the inhabited area is very limited in space, a selection against dispersal is very likely to occur [4,80]. It can be suspected that there was a significant reduction in numbers in the Knyszyn Forest around 2019 (after the first sampling), i.e., when the species was extirpated in Czerwony Bór, which, in turn, was probably largely related to extreme weather conditions. Therefore, both bottleneck and genetic drift resulting from isolation may explain the observed pattern of diversity and differentiation. Our studies show that the process of genetic impoverishment can progress very quickly, even within a decade, also affecting the frequency of alleles. As a result, genetic differentiation between the Knyszyn Forest and Czerwony Bór has clearly increased over a period of ten years. Here, it should be mentioned that *C. myrmidone* is bivoltine and, in some years, even a three-generation species. The first (spring) brood can be clearly less numerous than the summer one [4], so intra-seasonal breakdowns are a serious problem and a threat to the existence of the population both directly and indirectly.

### 4.2. Wolbachia in Colias Myrmidone

The presence of *Wolbachia* was reported in *C. myrmidone* for the first time, and all infected individuals shared the Cm*COI*-2 haplotype. Our screening indicated a 100% prevalence in this genotype, whereas in the case of the most common and widespread Cm*COI*-1 haplotype, we did not record any cases of infection. The observed pattern can be easily explained by unidirectional cytoplasmic incompatibility, i.e., males infected with *Wolbachia* are not able to produce functional zygotes with uninfected females or those that do not host the same *Wolbachia* strain. Females, on the contrary, if infected, produce offspring regardless of their mate’s infection status [36,81]. *Wolbachia* is transmitted maternally, but nuclear gene flow between mtDNA lineages is not affected. This is consistent with our data indicating no differentiation in *EF-1α* between infected and uninfected genotypes. However, with the intermediate frequency of the *Wolbachia* infection, i.e., when not all individuals are infected, the patterns of infection can be surprising, e.g., in the case of *Colias mongola/C. tamerlana* when *Wolbachia* was exclusively detected in one haplogroup and was restricted to (not all) females randomly found in the geographically remote populations [82]. On the contrary, for two congeneric species, i.e., *C. croceus* and *C. erate,* studies in Slovakia indicate 100% infection [83].

The *Wolbachia* strain identified in our sample has been recorded so far only in an unrelated butterfly species—*Pseudophilotes bavius* [58]. Because either the origin of the infection or the impact on the host was not resolved by that study and remains unknown, it would be interesting to perform a detailed comparative examination of both species sharing the same *Wolbachia* sequence. The only identified strain in the population of any *Colias* species in Europe, i.e., ST141 belonging to supergroup B, exhibits a perfect vertical transmission and is known to induce strong cytoplasmic incompatibility when different infection status or different strains of a male and a female can cause the death of developing embryos [35].

The presence of a strain other than that previously detected in *Colias* spp., combined with the fact that the only infected haplotype (Cm*COI*-2) differs from the most common haplotype in the Polish population by a relatively high number of substitutions, and in phylogenetic analyses occupies a position in a separate part of the tree (close to *C. wisskoti*), which may suggest the transmission of the endoparasite by interspecies hybridization [84]. This concept, however, requires confirmation by further studies.

It is more puzzling that the prevalence of the Cm*COI*-2 haplotype and, therefore, the prevalence of infected genotypes was clearly, although incompletely, female-biased in the Knyszyn Forest (26.9% females and only 6.1% males), while the sex ratios in the studied metapopulation were fairly well balanced, as estimated during intensive mark–release–recapture performed in the same year [4]. Such cases are very rare, and the mechanisms behind this phenomenon, as well as their consequences, are insufficiently understood [85]. Narita et al. [86] suggest that a single strain of *Wolbachia* may induce two distinct reproductive manipulations in the same host. Additionally, complete sex biases resulting from male killing or feminization caused by *Wolbachia* are well documented for lepidopterans [87,88,89].

We did not find the “*Wolbachia*-infected” haplotype (Cm*COI*-2) in material collected a decade earlier; however, it should be noted that, either in the Knyszyn Forest and Czerwony Bór, a significantly smaller number of individuals was sampled. Moreover, information on the sex of most individuals was missing (because it was regarded as redundant at that time by a collector). Sampling for genetic studies is frequently male-biased due to the higher catchability of this sex, which is related to more conspicuous behavior. Studies often tend to sample material from males or simply collect worn males (see, e.g., [90]), as they are considered less important for populations than gravid females. This concern is often even more emphasized in the case of scarce and/or endangered species. Our experience shows that complete information and/or unbiased sampling would be recommended. We suspect that Cm*COI*-2 was present in the Knyszyn Forest in 2014, but this issue cannot be resolved in relation to Czerwony Bór, taking the sampling constraints into consideration. Samples were collected from a dozen or so individuals and mostly males, among which the “*Wolbachia*-infected” haplotype (Cm*COI*-2) was rare. The same could be applied to all *C. myrmidone* populations sampled for barcoding libraries to date, especially those from which there have been sequences from only 11 individuals available until now. The presence of *Wolbachia* in *C. myrmidone* individuals, for which sequences were deposited in GenBank, remains unknown; it would also be especially interesting to obtain insight into populations from Russia, where Cm*COI*-2 was previously detected.

Mitochondrial DNA not being neutral, as was considered in the past, may influence some functional traits, including adaptation to climatic conditions [91]. Variation in mtDNA sequences may, e.g., potentially counterbalance some environmental changes, and adaptive mutations spread, subsequently producing selective sweeps in populations [92]. Therefore, the presence of both infected and uninfected genotypes (as detected in the Knyszyn Forest) could be beneficial for the population in some way, but further studies are needed. The focal population history of infection is unknown, and it is not possible to judge whether the *Wolbachia* prevalence rate, estimated at 14.4% for samples collected in 2022, is stable among seasons.

The only identified strain (ST141 belonging to supergroup B) in the population of any *Colias* in Europe exhibits perfect vertical transmission, and it is known to induce strong cytoplasmic incompatibility when different infection status or different strains of a male and a female can cause death of developing embryos [83]. Studies of *C. erate* in Japan indicate that *Wolbachia* seems to have positive effects on host fitness, as assessed by the survival rates of the infected brood [93].

### 4.3. Differentiation Between Colias myrmidone and Colias caucasica

Interestingly, the most common Cm*COI*-1 haplotype detected in *C. myrmidone* samples was also shared with *Colias caucasica balcanica*. Searching GenBank, we found that 16 of 18 sequences were identical to Cm*COI*-1, and they were detected in individuals from Macedonia and Serbia. Only two specimens of *C. caucasica* from Greece had distinct and unique haplotypes that differed significantly (14 substitutions) from each other, with one being very close to Cm*COI*-1 (differing by a single substitution) and another one differing from Cm*COI*-2 by two substitutions. This may suggest that two different haplogroups may also occur in the Balkans.

Barcode sharing is reported for 69 butterfly species (15%) occurring in Europe and may be explained by biological processes (e.g., hybridization resulting in introgression or incomplete lineage sorting), as well as uncertainties related to taxonomy or simply the misidentification of sampled material [29]. Then, among *Colias* butterflies, introgression following hybridization may explain the observed barcode sharing as seen in, e.g., *C. behrii* and *C. meadii* in North America [94].

In the case of *C. myrmidone/C. caucasica*, incomplete lineage sorting cannot be excluded. It seems unlikely that barcode sharing is related to *Wolbachia* infection since we did not find infected individuals within the Cm*COI*-1 haplogroup. Recent analyses based on sequences of multiple genes indicate that *C. myrmidone* and *Colias caucasica* Staudinger, 1871 are sister species, separated by ~1.2 Ma [25]. However, only two individuals (from Russia and Georgia, respectively) were included. Further studies are desired, especially taking into consideration the ca. 1500 km disjunction between nominotypical *C. caucasica* occupying mountains of eastern Turkey [95] and part of the Caucasus [96], as well as the nearest populations of *C. caucasica balcanica,* which is considered as an endemic Balkan subspecies ranging from Croatia to Greece [97,98,99,100,101,102,103]. Initially, it was considered as a mountain variant of *C. myrmidone,* a subspecies of *C. myrmidone*, or a distinct species, *Colias balcanica* [104], and the present status was evaluated by Wegner [105]. Generally, *C. caucasica* resembles *C. myrmidone,* but it is somewhat larger, and in males, the ground color of the wing upper side is a deeper orange [106]; however, both taxa share the same configuration of UV pattern on their wings [107]. *Colias caucasica* inhabits mostly alpine grasslands (850–2300 m) and uses local *Chamaecytisus* species (e.g., *Ch. hirsutus*) as larval food plants. Contrasting to *C. myrmidone*, there is a single generation per year in nature, but the second brood is easily produced in captive rearing [106], which suggests that some traits may be adapted to harsh environmental conditions at high altitudes.

There are approximately 90 described *Colias* species—the vast majority of which are Holoarctic ones—which makes this genus one of the largest among Pieridae [108,109]. Although they are quite spectacular butterflies with a long history of both collecting and studies, there are still a lot of taxonomic ambiguities resulting from intraspecies variation in wing pattern. At the same time, genitalia structures are largely uniform in this genus and do not possess reliable diagnostic features contrasting to most other lepidopterans. Moreover, allopatric populations with similar phenotypes were traditionally treated as separate species. Many weakly supported subspecies have already been synonymised [110]. Recent studies have investigated the conspecificity of *C. mongola* and *C. tamerlana*, enigmatic taxa in which *COI* haplotypes display four strongly supported lineages while nuclear markers demonstrate a very shallow divergence [82].

In particular, the case of *C. myrmidone* and *C. caucasica,* including *C. c. balcanica,* clearly shows that an ultimate taxonomic decision requires extensive morphological, ecological, genetic, and/or molecular evidence. We are unanimous with Dincă et al. [29] that further research is recommended and simultaneously emphasizes the need for extensive analyses on *Wolbachia* prevalence in populations of *C. myrmidone*/*C. caucasica*, as *Wolbachia*-mediated mitochondrial introgressions may obscure taxonomy [19,111,112].

### 4.4. Conservation Implications

The last Polish population retained a relatively high genetic diversity, although we also found signs of recent deterioration. To counteract this process, it is vital to conserve a network of habitats to minimize the probability of serious bottlenecks resulting from both abiotic and biotic (e.g., parasitoids) factors affecting seasonal abundance. However, to increase genetic diversity, assisted gene flow should be considered, i.e., exchanging individuals with other extant European populations. Such actions should be taken very carefully and proceeded by genetic studies to avoid negative effects related to *Wolbachia*.

The issue of evolutionary relationships between *C. myrmidone* and *C. caucasica balcanica* also remains to be resolved. Final conclusions will be important from a conservation point of view. If *Colias caucasica balcanica* is not distinct from *C. myrmidone*, the range of the latter in Europe would actually be wider, and the Balkan EU countries (Bulgaria, Croatia, and Greece) will receive a new Natura 2000 species because *C. caucasica* is not listed in the Habitats Directive.

## Figures and Tables

**Table 1:** Distribution of haplotypes and basic indicators of genetic diversity in the investigated populations of *C. myrmidone*, as estimated on the basis of a polymorphism of a (587 bp) fragment of mtDNA *COI* and absolute numbers of particular haplotypes in populations. *N*—sample size; *H*—number of haplotypes; *h*—haplotype diversity; *π*—nucleotide diversity; *k*—mean number of nucleotide differences among haplotypes; *SSD*—sum of squared deviations.

	Knyszyn Forest			Czerwony Bór	All
Year	2014	2022	2014–2022	2014	2014–2022
*N*	12	125	137	22	159
*H*	1	4	4	1	4
*h*	0.00	0.276	0.255	0.00	0.224
*π*	0.00	0.00556	0.00514	0.00	0.00452
*k*	0.00	3.262	3.018	0.00	2.652
	**Tajima’s *D* test**				
*D*	–	0.470	0.280	–	-0.0062
*p*	–	0.651	0.583	–	0.514
	**Fu’s *Fs* test**				
*F*s	–	8.544	8.062	–	7.277
*p*	–	0.999	0.990	–	0.985
	**Raggedness index**				
*r*	–	0.6035	0.6191	–	0.6466
*p*	–	0.556	0.602	–	0.658
	**SSD**				
*SSD*	–	0.0838	0.0724	–	0.0566
*p*	–	0.035	0.034	–	0.019
	**Frequency**				
Cm*COI*-1	1.000	0.840	0.854	1.000	0.87
Cm*COI*-2	–	0.144	0.131	–	0.11
Cm*COI*-3	–	0.008	0.007	–	0.01
Cm*COI*-4	–	0.008	0.007	–	0.01

**Table 2:** Distribution of haplotypes and basic indicators of genetic diversity in the investigated populations of *C. myrmidone* in Poland, as estimated on the basis of a polymorphism of a (581 bp) fragment of the nuclear *EF-1α* gene and absolute numbers of particular haplotypes in populations. *N*—sample size; *H*—number of haplotypes; *h*—haplotype diversity; *π*—nucleotide diversity; *k*—mean number of nucleotide differences among haplotypes; *SSD*—sum of squared deviations.

Locality	Knyszyn Forest			Czerwony Bór	All
Year	2014	2022	2014–2022	2014	2014–2022
*N*	11	124	135	16	151
*H*	6	11	14	9	18
*h*	0.855	0.571	0.600	0.925	0.646
*π*	0.00363	0.00157	0.00175	0.00485	0.00210
*k*	2.10909	0.91303	1.01438	2.81667	1.21996
	**Tajima’s *D* test**				
*D*	−0.94257	−0.38639	−0.91187	−0.24378	−1.33313
*p*	0.359	0.338	0.269	0.421	0.085
	**Fu’s *Fs* test**				
*F*s	−1.419	−6.502	−8.109	−2.779	−14.751
*p*	0.119	0.001	0.001	0.066	0.001
	**Raggedness index**				
*r*	0.0614	0.0728	0.0621	0.042	0.0483
*p*	0.658	0.765	0.757	0.468	0.791
	**SSD**				
*SSD*	0.0096	0.0077	0.0074	0.0061	0.0068
*p*	0.623	0.555	0.569	0.482	0.594
	**Frequency**				
Cm*EF*-1	0.364	0.645	0.622	0.188	0.58
Cm*EF*-2		0.065	0.059		0.05
Cm*EF*-3		0.073	0.067		0.06
Cm*EF*-4	0.182	0.024	0.037		0.03
Cm*EF*-5	0.091	0.113	0.111	0.188	0.12
Cm*EF*-6	0.091	0.016	0.022	0.063	0.02
Cm*EF*-7		0.008	0.007		0.01
Cm*EF*-8		0.016	0.015		0.01
Cm*EF*-9		0.024	0.022	0.063	0.03
Cm*EF*-10		0.008	0.007		0.01
Cm*EF*-11		0.008	0.007		0.01
Cm*EF*-12	0.182		0.015		0.01
Cm*EF*-13	0.091		0.007		0.01
Cm*EF*-14				0.063	0.01
Cm*EF*-15				0.125	0.01
Cm*EF*-16				0.125	0.01
Cm*EF*-17				0.125	0.01
Cm*EF*-18				0.063	0.01

## Data Availability

All the analyzed DNA sequences are available via the GenBank links provided.

## Supplementary Materials

The following supporting information can be downloaded at: https://www.mdpi.com/article/10.3390/insects16020220/s1, Table S1: Data summary for samples of *Colias myrmidone* collected in Poland and used in this study; Table S2: Data summary for the *COI* sequences of *Colias myrmidone* and *C. caucasica* available in GenBank and used in this study.
